# Iron and folic acid supplementation adherence among pregnant women attending antenatal care in North Wollo Zone northern Ethiopia: institution based cross-sectional study

**DOI:** 10.1186/s13104-019-4142-2

**Published:** 2019-03-05

**Authors:** Asmamaw Demis, Biftu Geda, Tadesse Alemayehu, Haimanot Abebe

**Affiliations:** 1 Department of Nursing, Faculty of Health Sciences, Woldia University, P.O.BOX:400, Woldia, Ethiopia; 2 School of Nursing and Midwifery, College of Health and Medical Sciences, Haramaya University, Haramaya, Ethiopia; 3 Department of Midwifery, College of Health and Medical Sciences, Wolkitie University, Wolkitie, Ethiopia

**Keywords:** Adherence status, Iron and folic acid, Pregnant women

## Abstract

**Objectives:**

The main aim of this study was to assess iron and folic acid supplementation adherence among pregnant mothers attending antenatal care in public health facilities of North Wollo Zone northern Ethiopia. An institution based quantitative cross-sectional study design was employed, on 422 pregnant women in North Wollo Zone, northern Ethiopia. Systematic random sampling and purposive sampling methods were used to select study participants for the quantitative and qualitative studies respectively.

**Results:**

The overall adherence status of pregnant women attending antenatal clinic was found to be 43.1% (95% CI, 38.6%–48.1%). Obtained counseling about iron and folic acid supplementation (AOR = 2.93, 95% CI 1.43–6.03), having four or more antenatal care visit (AOR = 2.94, 95% CI 1.39–6.21), early registration time (AOR = 3.04, 95% CI 1.85–5.01), good knowledge of anemia (AOR = 2.25, 95% CI 1.32–3.82) and good knowledge of IFAS (AOR = 2.47, 95% CI 1.47–4.16) were statistically and positively associated with pregnant mothers adherence to iron and folic acid supplementation.

**Electronic supplementary material:**

The online version of this article (10.1186/s13104-019-4142-2) contains supplementary material, which is available to authorized users.

## Introduction

Adherence to iron and folic acid is crucial for the prevention of birth defects and anemia during pregnancy [1]. Anemia is a global public health problem affecting majorly developing countries with major consequences for human health as well as social and economic development [2]. Globally, anemia affects 1.62 billion people, which corresponds to 25% of the population and approximately half of all anemia can be attributed to iron deficiency [3]. It is estimated that 38% of pregnant women worldwide are anemic with highest in Africa followed by South East Asia which accounts for 62.3% and 53.8% respectively [4, 5]. In Ethiopia prevalence of anemia among women aged 15–49 was 24% and 29% pregnant women were anemic which suggests that anemia is a major public health problem [6]. Different studies conducted in Ethiopia also showed that the prevalence of anemia among pregnant women were ranged from 21 to 54% [7–12]. The World Health organization targeted to reach a 50% reduction of anemia in women of reproductive age group by 2025 [13]. Iron and folic acid supplementation is the most widely employed strategy to alleviate iron deficiency, iron deficiency anemia and neural tube defects both globally and nationally [14, 15]. In Ethiopia nutrition is integrated in the health sector transformation plan in the form of micronutrient interventions to prevent the occurrence of anemia and improve the nutritional status of mothers during pregnancy [16]. Pregnant women are at particular risk of iron and folic acid deficiency due to their increased requirements. World health organization and National guideline recommend all pregnant women should receive a daily dose of 60 mg iron + 400 μg folic acid for 6 months and additional 3 months in setting where anemia prevalence is high, to prevent maternal anemia, puerperal sepsis, low birth weight (LBW) and preterm birth [14, 15].

Iron deficiency is one of the most prevalent nutrient deficiencies in the world, affecting more than 2 billion of the world’s population and causing an estimated 20% of maternal deaths [5]. Globally, 70% of women [17] and 41% in Nepal [18] didn’t took iron and folic acid supplements during pregnancy. In Ethiopia, nationally only 5% of pregnant women took IFAS for greater than 90 days but 58% did not take any iron-folic acid tablets during their most recent pregnancy [6]. A meta-analysis report showed that antenatal use of iron and folic acid supplement could eliminate 50% of iron deficiency anemia in pregnant women and also there is 19% reduction in the risk of low birth weight [19]. Early neonatal death was reduced by 57% in Nepal [18], 45% in Pakistan and 39% in Indonesia [20]. Adherence to iron and folic acid during pregnancy increases productivity and prevents iron deficiency anemia during pregnancy which reduces the risk of hemorrhage, sepsis and maternal mortality [21]. Poor adherence has a negative consequence on levels of energy and productivity, cognitive and physical development and immune function [22]. Similarly, iron deficiency anemia during pregnancy has fetal and neonatal risks, which include miscarriage, stillbirths, prematurity, low birth weight, congenital anomalies and perinatal mortality [23, 24].

Recent studies on the topic suggest that there were a number of reasons for non-adherence to IFAS including inadequate supplies [24], poor quality tablets [25, 26], lack of access to or use of prenatal care, and poor monitoring of the problem [27, 28]. Despite having many studies done elsewhere and efforts made to reduce iron deficiency anemia, there is a paucity of data concerning adherence status in Ethiopia particularly in North Wollo Zone of Ethiopia information about adherence to iron and folic acid supplementation and its associated factors are lacking. Therefore, this study assessed adherence status and identified factors associated with IFAS using quantitative studies in North Wollo Zone, northern Ethiopia.

## Main text

### Methods

#### Study setting and design and period

Institution-based quantitative cross-sectional study design was conducted from January 29 to March 16, 2018 at North Wollo Zone, which is located at Amhara National Regional State, at the northern part of Ethiopia. The zone is located about 521 km from Addis Ababa, the capital city of Ethiopia.

#### Sample size determination and sampling procedure

The sample size was determined by a computer based on Epi info 7 software Stat Cal using double proportion formula with the assumptions of 95% CI, 5% margin of error, 80% power and exposed to unexposed ratio 1 and 10% non-response the maximum sample size was 427. In Woldia Town Administration, there were two public health centers and one referral hospital. All of those facilities were included in the study. The average estimated number of pregnant women attending the antenatal clinic in each antenatal facilities for 3 months was taken. Accordingly, the calculated sample was distributed into these health facilities proportional to the size of women attending ANC in these 3 months’ time. Finally, a systematic random sampling was used to include participants in the study.

#### Data collection procedure

Data were collected using a pretested and structured interviewer administered questionnaire. The questionnaire was prepared in English and translated to Amharic, then back to English to check for its consistency. The reliability of the tool for knowledge related item was checked using Cronbach’s alpha reliability test, which was 0.79, which showed the consistency of the questionnaire. To assure the data quality, diploma nurses and B.Sc. public health professional were recruited as data collectors and supervisor, respectively. In addition, training regarding the study objectives and data collection process was given for data collectors and supervisor for 2 days. Moreover, the questionnaire was pretested among 5% of the sample size at Kobo primary hospital. Furthermore, intensive supervision was done by supervisor and principal investigators throughout the data collection period.

#### Data processing and analysis

The quantitative data was coded, cleaned, edited and entered into Epi data version 4.2 and exported to SPSS window version 24 for analysis. Descriptive results were presented using tables and figures. Model fitness was checked using a Hosmer–Lemeshow goodness-of-fitness test. Crude odds ratios with their 95% confidence intervals were estimated in the bi-variable logistic regression analysis to assess the association between each independent variable and outcome variable. All variables with P ≤ 0.25 in the bivariate analysis were included in the final model of multivariate analysis in order to control all possible confounders. Adjusted odds ratio with 95% CI was estimated to identify the factors associated with adherence status using multivariable logistic regression analysis. Level of statistical significance was declared at P-value < 0.05.

### Results

#### Socio-demographic characteristics

A total of 422 study participants were involved in the study. The mean age of study participants was 26.38 (± 4.26 SD) years. All most all, 413 (97.9%) of the study participants were married, 299 (70.9%) were Orthodox by religion and 392 (92.9%) were Amhara by ethnicity. One hundred sixteen (27.5%) were at college level and 190 (45%) were housewives. The majority, 367 (87.0%) of the respondents were from urban residents and 242 (57.3%) had 1**–**3 family size (Additional file 1: Table S1).

#### Obstetrics and health related characteristics

The mean gestational age of the pregnant women during the current visit was 29.14 (SD ± 5.68) weeks and 223 (52.8%) of them were in third trimester. The mean gestational age of the pregnant women during their first visit was 14.87 (SD ± 4.23) weeks and 241 (57.1%) were early registered for ANC. Regarding gravidity and parity, more than three-fifth (67.3%) of women were multigravida and 187 (44.3%) were primiparous. This study showed that only 8.5% of women had history of anemia in the current pregnancy. Concerning the utilization of antenatal care, more than half, 235 (55.7%) of respondents had two antenatal visits and 128 (30.3%) had three ANC visits (Table 1).

**Table 1:** Obstetrics and health related characteristics of pregnant women attending antenatal care in North Wollo Zone, northern Ethiopia, 2018

Variables	Adherence		Frequency (%)
	Yes (%)	No (%)	
*Gravidity*			
Primigravida	62 (44.6)	77 (55.4)	139 (32.7)
Multigravida	120 (42.4)	163 (57.6)	283 (67.3)
*Parity*			
Nulliparous	63 (45.3)	76 (54.7)	139 (32.9)
Primiparous	85 (45.4)	102 (54.6)	187 (44.3)
Multiparous	34 (35.4)	62 (64.6)	96 (22.8)
*Birth spacing in years (n* = *283)*			
1–2	21 (36.8)	36 (63.2)	57 (20.1)
≥ 3	98 (43.4)	128 (56.6)	226 (79.9)
*ANC visit*			
2 visits	95 (40.4)	140 (59.6)	235 (55.7)
3 visits	43 (33.6)	85 (66.4)	128 (30.3)
≥ 4 visits	44 (74.6)	15 (25.4)	59 (14)
*First registration time for ANC*			
Early registration	130 (53.9)	111 (46.1)	241 (57.1)
Late registration	52 (28.7)	129 (71.3)	181 (42.9)
*Current visit trimester*			
Second trimester	75 (37.7)	124 (62.3)	199 (47.2)
Third trimester	107 (48.0)	116 (52.0)	223 (52.8)
*Medical illness other than anemia*			
Yes	10 (29.4)	24 (70.6)	34 (8.1)
No	172 (44.3)	216 (55.7)	388 (91.9)
*History of anemia during previous pregnancy (n* = *283)*			
Yes	22 (62.8)	13 (37.2)	35 (12.4)
No	96 (38.7)	152 (61.3)	248 (87.6)
*Current anemia status*			
Anemic	19 (52.8)	17 (47.2)	36 (8.5)
Non Anemic	163 (42.2)	223 (57.8)	386 (91.5)
*Haemoglobin recorded gestational week*			
First trimester	70 (53.8)	60 (46.2)	130 (30.8)
Second and third trimester	112 (38.3)	180 (61.7)	292 (68.5)

#### Knowledge status of respondents on Anemia and Benefit of IFAS

Majority, 368 (87.2%) of the respondents have ever heard about anemia. About 216 (51.2%) of the respondents had good knowledge about anemia whereas 48.8% had poor knowledge of anemia. Regarding knowledge on the benefit of IFAS 242 (57.3%) of the respondents had good knowledge whereas 42.7% had poor knowledge about iron and folic acid supplementation.

#### Health facility related characteristics

Of the total respondents, 176 (41.7%) said that, it took them 30 min or less to reach the nearest health facility from their place of residence. Regarding waiting time, 264 (62.6%) of respondents wait less than or equal to 30 min in the health institutions (Table 2).

**Table 2:** Health facility related characteristics of pregnant women attending antenatal care in North Wollo Zone, northern Ethiopia, 2018

Variables	Adherence		Frequency (%)
	Yes (%)	No (%)	
*Facility distance (min)*			
≤ 30	76 (43.2)	100 (56.8)	176 (41.7%)
> 30	106 (43.1)	140 (56.9)	246 (58.3%)
*Waiting time (min)*			
≤ 30	112 (42.4)	152 (57.6)	264 (62.6%)
> 30	70 (44.3)	88 (55.7)	158 (37.4%)
*Obtain counseling on IFA*			
Yes	169 (48.0)	183 (52.0)	352 (83.4%)
No	13 (18.6)	57 (81.4)	70 (16.6%)
*Encountered shortage of supplement*			
Yes	24 (30.8)	54 (69.2)	78 (18.5%)
No	158 (45.9)	186 (54.1)	344 (81.5%)
*Got IFAS free of charge*			
Yes	166 (44.9)	203 (55.1)	369 (87.4%)
No	16 (30.2)	37 (69.7)	53 (12.6%)

#### Adherence status to IFA supplementation

The overall adherence status (took IFA tablets for ≥ 4 days/week for the previous 1 month preceding the survey) of pregnant women attending antenatal clinics was 43.1%.

#### Factors associated with adherence to IFA supplementation

The covariates of this study were: mother’s education, residence, current anemia, getting IFA free of charge, ANC visit, encountered shortage of IFAS, first registration week, obtained counseling, anemia knowledge and knowledge of IFAS were candidate for multivariable model. In multivariable model obtaining counseling about IFAS, ANC visit, knowledge of anemia, knowledge of IFAS and first registration week were statistically associated with adherence to iron and folic acid supplementation.

Mothers who had obtained counseling about IFAS were almost three times more likely adhere to IFAS than those who had not obtained counseling (AOR = 2.93, 95% CI 1.43–6.03). Mothers who had four or more ANC visit were almost three times (AOR = 2.94, 95% CI 1.39–6.21) and who had early registration time were 3.04 times more likely to adhere (AOR = 3.04, 95% CI 1.85–5.01). Regarding knowledge of mothers, those who had good knowledge of anemia were 2.25 times (AOR = 2.25, 95% CI 1.32–3.82) and those who had good knowledge of IFAS were 2.5 times (AOR = 2.47, 95% CI 1.47–4.16) more likely adhere to IFAS as compared to those who had poor knowledge (Table 3).

**Table 3:** Factors associated with adherence to iron and folic acid supplementation among pregnant women attending antenatal care in North Wollo Zone, (COR and AOR) northern Ethiopia, 2018

Variables	Adherence		COR (95% CI)	AOR (95% CI)
	Yes (%)	No (%)		
*Mother educational status*				
No formal education	64 (38.3)	103 (61.7)	1	1
Primary (grade 1–8th)	24 (40.0)	36 (60.0)	1.07 (0.58–1.96)	1.39 (0.66–2.93)
Secondary and above	94 (48.2)	101 (51.8)	1.49 (0.98–2.28)	0.96 (0.58–1.59)
*Residence*				
Urban	173 (47.1)	194 (52.9)	4.55 (2.16–9.58)	2.34 (0.99–5.51)
Rural	9 (16.4)	46 (83.6)	1	1
*Getting IFA tablet free of charge*				
Yes	166 (44.9)	203 (55.1)	1.89 (1.01–3.52)	2.12 (0.97–4.51)
No	16 (30.2)	37 (69.8)	1	1
*Current anemia status*				
Anemic	19 (52.7)	17 (47.3)	1.53 (0.77–3.03)	1.96 (0.85–4.49)
Non anemic	163 (42.2)	223 (57.8)	1	1
*Obtain counseling on IFA*				
Yes	169 (48)	183 (52)	4.04 (2.14–7.66)	2.93 (1.43–6.03)*
No	13 (18.6)	57 (81.4)	1	1
*Encountered shortage of IFA*				
Yes	24 (30.8)	54 (69.2)	0.52 (0.31–0.88)	0.74 (0.38–1.45)
No	158 (45.9)	186 (54.1)	1	1
*Frequency of ANC visits*				
≥ 4 visits	44 (74.6)	15 (25.4)	4.78 (2.56–8.92)	2.94 (1.39–6.21)**
< 4 visits	138 (38)	225 (62)	1	1
*Time of registration*				
Early (< 16 weeks)	130 (53.9)	111 (46.1)	2.90 (1.93–4.37)	3.04 (1.85–5.01)***
Late (≥ 16 weeks)	52 (28.7)	129 (71.3)	1	1
*Knowledge of anemia*				
Good knowledge	129 (59.7)	87 (40.3)	4.28 (2.83–6.48)	2.25 (1.32–3.82)*
Poor knowledge	53 (25.7)	153 (74.3)	1	1
*Knowledge of IFAS*				
Good knowledge	139 (57.4)	103 (42.6)	4.30 (2.80–6.59)	2.47 (1.47–4.16)****
Poor knowledge	43 (23.9)	137 (76.1)	1	1

Significant at: * P = 0.003, ** P = 005, *** P < 0.001, **** P = 0.001, 1 = reference

### Discussions

The overall pregnant women adherence to iron and folic acid supplementation was 43.1%. This finding is low and as a result it might increase health care costs, iron deficiency anemia during pregnancy with poor maternal and fetal outcome and poor physical and cognitive development. This finding was in line with the results of studies conducted in other studies in Ethiopia Tigray [27] and South [29]. But it was higher than the study conducted in Kenya [30], Afar, Ethiopia [34] and Western Amhara, Ethiopia [28]. This difference may be due to increase pregnant women knowledge about anemia and iron folic acid supplementation, (as majority of the respondents in this study were from urban), difference in socio demographic characteristics of the respondents and the time gap between these studies. But it was lower than the study conducted from India [25], Senegal [31], eight rural district of Ethiopia [32] and Bench Maji Zone, Ethiopia [33]. This might be due to the difference in geographic location because mothers who came from high altitude, malaria area and anemic area are more likely adhered and most of the study uses community based cross sectional study design.

Pregnant women who had obtained counseling about IFAS were almost three times more likely adhered compared to those women who had not obtained counseling. This is in line with the study conducted in Senegal [31], Afar, Ethiopia [34], South, Ethiopia [29] and Bench Maji Zone, Ethiopia [33]. This might be due to the fact that women who had obtained counseling may understand the benefit of taking the supplement as ordered at the right time for themselves as well as for the growing fetus which makes them adhered to the prescribed supplement. In addition, it might be explained by the potential effect of counseling on self-care behaviors and managing side effects, the more counseled on IFAS benefit are more likely to psychologically tolerating the side effects during pregnancy, thus, the more likely to adhere with the prescription and/or recommendations.

Pregnant mothers who are registering for ANC early have the chance of more ANC visits throughout their pregnancy. This will exposed them for more knowledge regarding iron deficiency anemia and benefit of iron and folic acid supplementation from other pregnant women as well as health care providers during their long ANC visits.

Adherence status was better observed in those pregnant women’s who had four or more ANC visits which is similar to other studies conducted in Senegal [31], South, Ethiopia [29] and Tigray, Ethiopia [27]. This might be due to health care providers in charge of ANC service may counsel pregnant women on the benefit of taking the supplement at the right time and dose by discussing with adherence benefit and consequence of non-adherence for the mother and the fetus which ultimately improves the adherence status of pregnant women to the supplement.

Having good knowledge of anemia was significantly and positively associated with pregnant women’s adherence to iron and folic acid supplementation in which adherence was more likely among pregnant women’s who were knowledgeable for anemia. This finding is similar with the studies conducted in South, Ethiopia [29], Bench Maji Zone, Ethiopia [33], and Western Amhara, Ethiopia [28]. The probable reason could be due to the fact that knowledge helps women to have a good perception on prevention and treatment of anemia by taking iron-folate supplement during pregnancy.

Knowledge of iron and folic acid supplementation was also associated with women’s adherence to iron and folic acid supplementation in studies conducted in Korea [35], South, Ethiopia [29] and Western Amhara, Ethiopia [28]. Similarly, in this study being knowledgeable for iron and folic acid supplementation was an independent predictor for women’s adherence to iron and folic acid supplementation. This may be due to the fact that knowledge helps women to understand the benefit of taking the supplement and consequence of not taking the supplement to the mother and the fetus during pregnancy, labor and delivery. In addition, it might be explained by knowledge result in good perception of the women about prevention and treatment of anemia by taking iron and folic acid as prescribed and recommended by health care providers which in turn results in adherence to the prescribed supplement. Health executive bodies should strengthen the idea of client centered individualized care with provision of relevant and timely information during each ANC visit to improve mother’s knowledge and adherence status to iron and folic acid supplementation. The other researchers do further investigation to identify other factors by using pill count method and other study design like longitudinal study design.

### Limitations of the study

Due to the cross-sectional nature of this study, establishing a true cause and effect relationship between adherence status and associated factors would be impossible. This study might also suffer from recall bias.

## Additional file

**Additional file 1: Table S1.** Distribution of socio-demographic characteristics of pregnant women’s attending ANC in North Wollo Zone, northern Ethiopia, 2018.

## Abbreviations

ANC: antenatal care
CSA: Central Statistical Agency
EDHS: Ethiopian Demographic and Health Survey
FDRE: Federal Democratic Republic of Ethiopia
FMoH: Federal Ministry of Health
HSTP: Health Sector Transformation Plan
IFAS: iron and folic acid supplementation
NNP: National Nutrition Programme
SDG: sustainable development goals
SPSS: Statistical Package for Social Sciences
UNICEF: United Nations Children’s Fund
WHO: World Health Organizations
