# Supplementary material for: Iron and folic acid supplementation adherence among pregnant women attending antenatal care in North Wollo Zone northern Ethiopia: institution based cross-sectional study
Source: BMC Res Notes. 2019 Mar 5;12:107. doi: 10.1186/s13104-019-4142-2 (PMC6889669; doi:10.1186/s13104-019-4142-2)
Supplement: Supplementary file 1 — Additional file 1: Table S1. Distribution of socio-demographic characteristics of pregnant women’s attending ANC in North Wollo Zone, northern Ethiopia, 2018. [file 13104_2019_4142_MOESM1_ESM.docx]

Table S1: Distribution of socio-demographic characteristics of pregnant women’s attending ANC in North Wollo Zone, Northern Ethiopia, 2018.

| **Variables** | **Frequency (%)** |
| --- | --- |
| **Age** |  |
| 18-24 | 146(34.6) |
| 25-34 | 250(59.2) |
| ≥ 35 | 26(6.2) |
| **Marital status** |  |
| Married | 413(97.9) |
| Others* | 9(2.1) |
| **Religion** |  |
| Orthodox | 299(70.8) |
| Muslim | 110(26.1) |
| Others** | 13(3.1) |
| **Ethnicity** |  |
| Amhara | 392(92.9) |
| Others*** | 30(7.1) |
| **Educational level of mother** |  |
| No formal education | 167(39.6) |
| Primary education | 60(14.2) |
| Secondary and above  **Occupation of mother** | 195(46.2) |
| House wife | 190(45.0) |
| Gov’t employee  Self-employee | 103(24.4)  68(16.1) |
| Farmer | 42(10.0) |
| Others**** | 19(4.5) |
| **Educational level** of **husband** (n=413) |  |
| No formal education | 95(23.0) |
| Primary education | 53(12.8) |
| Secondary and above | 265(64.2) |
| **Occupation of husband** (n=413) |  |
| Gov’t employee | 179(43.3) |
| Self-employee | 141(34.1) |
| Farmer | 58(14.1) |
| Others**** | 35(8.5) |
| **Family size** |  |
| 1-3 | 242(57.3) |
| ≥ 4 | 180(42.7) |
| **Place of residence** |  |
| Urban | 367(87.0) |
| Rural | 55(13.0) |

*Other*: Divorced. Widowed, Single, Other**: protestant, catholic, other***: Afar, Oromo, Tigray, Other****: student, daily laborer, private employee*
